# The association between metabolic syndrome and the risk of urothelial carcinoma of the bladder: a case-control study in China

**DOI:** 10.1186/s12957-015-0631-5

**Published:** 2015-08-07

**Authors:** Sheng Xu, Gui-Ming Zhang, Feng-Ju Guan, Da-Hai Dong, Lei Luo, Bin Li, Xiao-Cheng Ma, Jun Zhao, Li-Jiang Sun

**Affiliations:** Department of Urology, The Affiliated Hospital of Qingdao University, Qingdao, Shandong China; Department of Surgery, The Affiliated Hospital of Qingdao University, Qingdao, Shandong China; Department of Urology, The Second Affiliated Hospital of Xi’an Jiaotong University, Xi’an, Shanxi China

**Keywords:** Urothelial carcinoma of the bladder, Metabolic syndrome, Diabetes mellitus, Hypertriglyceridemia, Epidemiology

## Abstract

**Background:**

Evidence of the association of metabolic syndrome (MetS) with cancer risk is accumulating. However, uncertainties still exist as to the link of MetS with bladder cancer. This study aimed to assess the relationship between MetS and the risk of urothelial carcinoma of the bladder (UC) in a Chinese population.

**Methods:**

We retrospectively analyzed clinicopathological data of 972 newly diagnosed UC patients and 1098 cancer-free controls matched to the cases by age and gender. Odds ratios (ORs) and 95 % confidence intervals (CIs) were calculated using unconditional logistic regression in both unadjusted and adjusted models.

**Results:**

MetS was not significantly associated with the overall UC risk (*p* = 0.08). However, a significant association of MetS with UC was observed in female patients (*p* = 0.006). Diabetes mellitus (crude OR 1.339, 95 % CI 1.079–1.662, *p* = 0.008; adjusted OR 1.767, 95 % CI 1.308–2.386, *p* < 0.001) and hypertriglyceridemia (crude OR 1.245, 95 % CI 1.018–1.522, *p* = 0.033; adjusted OR 1.254, 95 % CI 1.020–1.542, *p* = 0.032) were significantly associated with UC risk. As the number of MetS components increased, the UC risk was elevated. Having three or more (versus zero) components of MetS was significantly related to risk of overall UC (OR 1.315; 95 % CI 1.006–1.719; *p* = 0.045) and non-muscle invasive bladder cancer (OR 1.354; 95 % CI 1.019–1.798; *p* = 0.037).

**Conclusions:**

The present study indicated a marginal association between MetS and UC risk, and a significant association with UC risk in female patients. The results need to be evaluated in large-scale prospective cohorts.

## Background

Urothelial carcinoma of the bladder (UC) is the most common malignancy of the urinary tract, ranking fourth in incidence in Western countries and first in China [1]. Both inherited and environmental factors, such as smoking, contact with aromatic amine compounds, and human papillomavirus infection, play vital roles in the carcinogenesis of UC [2–4]. However, these well-established risk factors cannot fully illuminate the comprehensive mechanisms of UC initiation and development.

Metabolic syndrome (MetS), a cluster of abnormal metabolic factors related to excess calorie intake and sedentary lifestyles, has become a global health issue with growing prevalence [5]. To date, different organizations have introduced several criteria for MetS, all of which are based on insulin resistance, a quantificational index that cannot be easily measured. MetS mostly features [6] obesity, diabetes mellitus, hypertension, and dyslipidemia, and is considered an important risk factor for cardiovascular diseases (CAD) [7]. Recently, evidence of a relationship between MetS and the risk of cancer is accumulating in diseases such as colorectal cancer, breast cancer, lung cancer, prostate cancer, and renal cell carcinoma [8–12]. However, only a few studies [13, 14] have examined the association of MetS with UC and drawn conflicting conclusions. A weak significant association was found between MetS and UC risk in men in Esposito’s [12] study. However, Cantiello et al. [15] found that MetS did not represent an independent risk factor in UC. Given the relative scarcity and inconsistency of existing data, our intention is to provide additional information on the relationship between MetS and UC.

## Methods

### Study subjects

We gathered the data of 972 patients with newly diagnosed, pathologically confirmed UC at the Department of Urology at the Affiliated Hospital of Qingdao University from June 2011 to March 2014 and at the Department of Urology at the Second Affiliated Hospital of Xi’an Jiaotong University from January 2013 to March 2014. Patients with non-urothelial carcinoma of bladder were excluded from the study. In addition, for patients who were re-admitted to the hospital due to tumor recurrence and/or distant metastasis, only the information of first admission was included. We recruited 1098 cancer-free controls that sought routine health checkups from June 2011 to March 2014 from the Affiliated Hospital of Qingdao University. They were matched to the cases by age and gender. Data on age, gender, diseases and medication history (hypertension, diabetes), smoking status, alcohol consumption, anthropometric measurements (weight, height), blood tests (circulating levels of glucose and lipid profiles), stage at diagnosis (TNM classification), and pathology grade were collected from medical records. The institutional research review boards of the two clinical centers approved our study protocols, and signed informed consent was obtained from all the participants.

### Exposure measures

Age, gender, smoking status, alcohol consumption, and MetS were introduced as risk factors. MetS is defined according to the standard of the Chinese Diabetes Society [16], which sets three of five of the following criteria as necessary: (1) overweight, defined as body mass index (BMI) ≥25 kg/m^2^; (2) hypertension, a self-report of a physician’s diagnosis of hypertension, or over 140 and 90 mmHg for systolic and diastolic blood pressure, respectively, on three consecutive occasions; (3) diabetes, defined as meeting any one of the following: elevated fasting glucose ≥7.0 mmol/L, use of drugs to retain a normal serum glucose level, or physician’s diagnosis; and (4) abnormal triglyceride (TG) or high-density lipoprotein (HDL) levels: hypertriglyceridemia, a serum TG level ≥150 mg/dL (1.7 mmol/L); and low HDL-cholesterol, defined as <40 mg/dL (1.03 mmol/L) (male) or <50 mg/dL (1. 3 mmol/L) (female).

### Statistical analyses

Statistical analyses were performed using SPSS 19.0 software (IBM Corporation, Somers, NY, USA). A *p* value <0.05 (two-tailed) was defined as statistically significant. To assess the differences among the dichotomous categorical variables, including overweight, smoking status (no or ever/current), alcohol consumption (no or yes), hypertension (no or yes), diabetes (no or yes), triglycerides (normal or abnormal), HDL (normal or abnormal), and MetS (no or yes), chi-squared tests were used. Crude and adjusted odds ratios (ORs) and 95 % confidence intervals (CIs) were estimated using logistic regression analysis models.

## Results

Of 972 UC cases and 1098 controls included in our study, the cases had a median age of 62 years and the controls had a median age of 63 years. No statistical significance was found in age, gender, alcohol consumption, or MetS between groups (Table 1). Smoking status was statistically associated with an elevated UC risk.

**Table 1:** Demographic and clinical characteristics of 972 UC cases and 1098 controls

Variables	Controls, n (%)	UC cases, n (%)	*P* value
Age, years			0.559
<45	111 (10.1)	86 (8.8)	
45-	196 (17.9)	159 (16.4)	
55-	314 (28.6)	289 (29.7)	
≥65	477 (43.4)	438 (45.1)	
Median (IQR)	62 (19)	63 (20)	
Gender			0.906
Male	914 (83.2)	811 (83.4)	
Female	184 (16.8)	161 (16.6)	
BMI, kg/m^2^			0.425
<25	610 (55.6)	523 (53.8)	
≥25	488 (44.4)	449 (46.2)	
Smoking status			0.005
No	697 (63.5)	558 (57.4)	
Ever/current	401 (36.5)	414 (42.6)	
Alcohol consumption			0.861
No	745 (67.9)	656 (67.5)	
Yes	353 (32.1)	316 (32.5)	
Hypertension			0.506
No	775 (70.6)	673 (69.2)	
Yes	323 (29.4)	299 (30.8)	
Diabetes mellitus			0.008
No	903 (82.2)	754 (77.6)	
Yes	195 (17.8)	218 (22.4)	
Lipid profiles, mg/dL			
Triglycerides			0.033
Normal (<150)	852 (77.6)	715 (73.6)	
Abnormal(≥150)	246 (22.4)	257 (26.4)	
HDL			0.158
Normal(≥40)	890 (81.1)	811 (83.4)	
Abnormal(<40)	208 (18.9)	161 (16.6)	
MetS			0.080
No	938 (85.4)	803 (82.6)	
Yes	160 (14.6)	169 (17.4)	
Stage at diagnosis			
Ta		567 (58.4)	
T_1_		220 (22.6)	
T_2_		98 (10.1)	
T_3_		57 (5.9)	
T_4_		16 (1.6)	
T_is_		14 (1.4)	
Pathology grade			
Papilloma		60 (6.2)	
LGMPUP		19 (2)	
LGPUC		421 (43.4)	
HGPUC		472 (48.6)	

^a^ *UC* urothelial carcinoma of bladder; *BMI* body mass index; *LGMPUP* low-grade malignant papillary urothelial papilloma; *LGPUC* low-grade papillary urothelial carcinoma; *HGPUC* high-grade papillary urothelial carcinoma

To further evaluate the link between individual components of MetS and UC risk, using univariate and multivariate logistic regression models, we found that diabetes (crude OR 1.339, 95 % CI 1.079–1.662; adjusted OR 1.767, 95 % CI 1.308–2.386) and hypertriglyceridemia (crude OR 1.245,95 % CI 1.018–1.522; adjusted OR 1.254, 95 % CI 1.020–1.542) were significantly related to UC risk. However, no association was observed between overweight, hypertension, or abnormal HDL levels and UC (Table 2).

**Table 2:** Logistic regression analysis of factors possibly related to UC risk

Factors	Overweight	Hypertension	Diabetes	Hypertriglyceridemia	Low-HDL-cholesterol
Controls, n (%)	488 (52.1)	323 (51.9)	195 (47.2)	246 (48.9)	208 (56.4)
UC cases, n (%)	449 (47.9)	299 (48.1)	218 (52.8)	257 (51.1)	161 (43.6)
*P* value	0.425	0.506	0.008	0.033	0.158
Crude OR (95 % CI)	1.073 (0.902–1.276)	1.066 (0.883–1.287)	1.339 (1.079–1.662)	1.245 (1.018–1.522)	0.849 (0.677–1.066)
Adjusted^a^ *P* value	0.456	0.577	0.000	0.032	0.085
Adjusted^a^ OR (95 % CI)	0.925 (0.754–1.135)	1.057 (0.871–1.282)	1.767 (1.308–2.386)	1.254 (1.020–1.542)	0.789 (0.626–1.119)

^a^Adjusted for age (continuous), smoking status, gender, BMI (continuous), hypertension, diabetes mellitus, hypertriglyceridemia, low HDL-cholesterol

Table 3 lists crude and adjusted ORs and 95 % CIs for overall UC risk, non-muscle invasive bladder cancer (NMIBC) risk, and invasive bladder cancer (IBC) risk according to the number of MetS components. There appeared to be a biologic gradient between the number of MetS components and the risk of UC. An increasing number of MetS components was associated with a higher probability of UC, NMIBC, and IBC diagnoses. In multivariate logistic regression analyses, having one component or two components of MetS (versus zero) was not related to an increased risk of overall UC, NMIBC, or IBC. However, having three or more (versus zero) components of MetS was significantly associated with a risk of overall UC (OR 1.315; 95 % CI 1.006–1.719; *p* = 0.045) and NMIBC (OR 1.354; 95 % CI 1.019–1.798; *p* = 0.037), but not IBC (OR 1.196; 95 % CI 0.744–1.924; *p* = 0.459).

**Table 3:** Logistic regression analysis of the number of cumulative MetS components and overall UC risk, non-muscle invasive bladder cancer (NMIBC) risk, and invasive bladder cancer (IBC) risk

		Number of metabolic risk factors				Dichotomously defined	
		0 components	1 component	2 components	≥3 components	MetS	No MetS
Overall UC	Controls (n)	355 (55.1)	363 (53.6)	220 (52.4)	160 (48.6)		
	UC cases (n)	289 (44.9)	314 (46.4)	200 (47.6)	169 (51.4)		
	*P* value	Ref	0.583	0.380	0.055		
	Crude OR (95 % CI)	Ref	1.063 (0.856–1.320)	1.117 (0.873–1.429)	1.297 (0.994–1.693)		
	Adjusted^a^ *P* value	Ref	0.537	0.398	0.045		
	Adjusted^a^ OR (95 % CI)	Ref	1.071 (0.862–1.331)	1.113 (0.869–1.425)	1.315 (1.006–1.719)		
NMIBC	Controls (n)	355 (60.9)	363 (57.9)	220 (56.3)	160 (53.7)	160 (53.7)	938 (58.6)
	UC cases (n)	228 (39.1)	264 (42.1)	171 (43.7)	138 (46.3)	138 (46.3)	663 (41.4)
	*P* value	Ref	0.289	0.150	0.040	0.116	Ref
	Crude OR (95 % CI)	Ref	1.132 (0.900–1.425)	1.210 (0.933–1.570)	1.343 (1.013–1.780)	1.220 (0.952–1.564)	Ref
	Adjusted^a^ *P* value	Ref	0.264	0.169	0.037	0.107	Ref
	Adjusted^a^ OR (95 % CI)	Ref	1.140 (0.906–1.436)	1.201 (0.925–1.559)	1.354 (1.019–1.798)	1.228 (0.957–1.577)	Ref
IBC	Controls (n)	355 (85.3)	363 (87.9)	220 (88.4)	160 (83.8)	160 (83.8)	938 (87.0)
	UC cases (n)	61 (14.7)	50 (12.1)	29 (11.6)	31 (16.2)	31 (16.2)	140 (13.0)
	*P* value	Ref	0.280	0.272	0.617	0.227	Ref
	Crude OR (95 % CI)	Ref	0.802 (0.537–1.198)	0.767 (0.478–1.231)	1.128 (0.704–1.806)	1.298 (0.850–1.983)	Ref
	Adjusted^a^ *P* value	Ref	0.362	0.309	0.459	0.162	Ref
	Adjusted^a^ OR (95 % CI)	Ref	0.829 (0.553–1.241)	0.781 (0.485–1.257)	1.196 (0.744–1.924)	1.356 (0.885–2.077)	Ref

*MetS* metabolic syndrome; *UC* urothelial carcinoma of bladder; *NMIBC* non-muscle invasive bladder cancer; *IBC* invasive bladder cancer

^a^Adjusted for age (continuous), smoking status, gender

We next analyzed the association of MetS, diabetes, and hypertriglyceridemia with UC risk in male and female patients. In female patients, MetS was significantly associated with increased UC risk (crude *p* = 0.006, adjusted *p* = 0.009); however, there were no statistically significant differences in male patients (Table 4). Neither diabetes nor hypertriglyceridemia was an independent risk factor of UC in female patients; Hypertriglyceridemia was an independent risk factor of UC only in male patients (Table 5).

**Table 4:** Logistic regression analysis of association of MetS and UC risk in male and female patients

		MetS	
		Yes	No
Male	Controls, n (%)	134 (14.7)	780 (85.3)
	UC cases, n (%)	127 (15.7)	684 (84.3)
	*P* value	0.563	Ref
	Crude OR (95 % CI)	1.081 (0.830–1.407)	Ref
	Adjusted^a^ *P* value	0.481	Ref
	Adjusted^a^ OR (95 % CI)	1.100 (0.844–1.434)	Ref
Female	Controls, n (%)	26 (14.1)	158 (85.9)
	UC cases, n (%)	42 (26.1)	119 (73.9)
	*P* value	0.006	Ref
	Crude OR (95 % CI)	2.145 (1.245–3.695)	Ref
	Adjusted^a^ *P* value	0.009	Ref
	Adjusted^a^ OR (95 % CI)	2.074 (1.200–3.586)	Ref

^a^Adjusted for age (continuous), smoking status

**Table 5:** Logistic regression analysis of association of diabetes mellitus, triglycerides and UC risk in male and female patients

Gender		Diabetes mellitus		Triglycerides	
		Yes	No	Yes	No
Male	Controls, *n* (%)	168 (18.4)	746 (81.6)	190 (20.8)	724 (79.2)
	UC cases, *n* (%)	174 (21.5)	637 (78.5)	202 (24.9)	609 (75.1)
	*p* value	0.110	Ref	*0.042*	Ref
	Crude OR (95 % CI)	1.213 (0.957–1.537)	Ref	1.264 (1.009–1.584)	Ref
	Adjusted^a^ *p* value	*0.001*	Ref	*0.038*	Ref
	Adjusted^a^ OR (95 % CI)	1.757 (1.268–2.434)	Ref	1.276 (1.014–1.604)	Ref
Female	Controls, *n* (%)	27 (14.7)	157 (85.3)	56 (30.4)	128 (69.6)
	UC cases, *n* (%)	44 (27.3)	117 (72.7)	55 (34.2)	106 (65.8)
	*p* value	*0.004*	Ref	0.460	Ref
	Crude OR (95 % CI)	2.187 (1.280–3.736)	Ref	1.186 (0.754–1.865)	Ref
	Adjusted^a^ *p* value	0.08	Ref	0.593	Ref
	Adjusted^a^ OR (95 % CI)	2.032 (0.918–4.500)	Ref	1.145 (0.697–1.880)	Ref

^a^Adjusted for age (continuous), smoking status, BMI (continuous), hypertension, diabetes mellitus, hypertriglyceridemia, low HDL-cholesterol

## Discussion

In our retrospective study, a notable association of MetS with UC risk in female patients was observed. Diabetes and hypertriglyceridemia were prevalent in UC patients in comparison with the controls, and hypertriglyceridemia was significantly associated with increased UC risk in male patients. In addition, with an increasing number of components of the MetS, the risk of overall UC, NMIBC, and IBC was elevated.

Although previous studies have shown a significant association of MetS with some common cancers [8–12], a few studies have focused on the relationship between MetS and UC. Recently, Esposito et al. [12] found a weakly significant association (*p* = 0.013) between MetS and the risk of UC in men. Moreover, Ozbek et al. [14] showed that patients with MetS had a significantly higher T stage and grade of UC. Nevertheless, Cantiello et al. [15] reported that MetS did not represent an independent risk factor in UC. In our study, there was no significant difference between MetS and the risk of UC in men, yet a significant association between MetS and UC risk was found in women. The discrepancies might be caused by the differences in race of the study participants and our relatively small sample sizes in female patients. Furthermore, the definition of MetS and pharmaceutical treatment and lifestyle modifications are different compared with those in developed Western societies. In our study, the database included only a Chinese population, and the results may not be generalized to other ethnic groups. As we know, incidence of MetS varies widely across populations. Some factors, such as gender and race, can influence the prevalence of MetS. Hence, further studies based on a large-scale population are warranted.

Wallner et al. [17] indicated that analyzing MetS as a single condition may be inadequate for uniting all the multiple MetS components to a single variable, which may confuse or obscure the independent impacts and interactions of each individual metabolic component on cancer. Therefore, we investigated each single component of MetS and the association with UC risk. Holick et al. [18] and Haggstrom et al. [13] found no association of overweight with UC. Conversely, Koebnick et al. [19] indicated that overweight was related to increased UC risk. Another two research groups [15, 20] found that UC patients with higher BMI had worse tumor characteristics. Interestingly, Goldbohm et al. [21] reported that overweight was a protective factor for UC. The relationship of hypertension with UC risk was likewise inconsistent. A Japanese case-control study [22] reported a lower risk of UC in men with hypertension. However, Haggstrom et al. [13] reported that higher blood pressure was consistently related to higher UC risk. In our study, both overweight and hypertension were not related to increased UC risk. In addition, diabetes was observed as an independent risk factor for UC in our study, which was in agreement with previous studies. Recently, a cohort research [23, 24] reported a significant association of diabetes with increased risk of bladder cancer, without distinguishing the gender. A meta-analysis conducted by Larsson et al. [25] demonstrated that high glucose levels were prevalent in UC patients. Research on the link of dyslipidemia and UC risk is scarce. In Haggstrom’s [13] study, there was no significant association between serum levels of cholesterol and TG and UC risk. However, the study conducted by Schatzkin et al. [26] showed an inverse association between cholesterol levels and UC. Our results showed that hypertriglyceridemia was an independent risk factor for UC risk in the total and male patients.

MetS was most commonly analyzed in a binary manner in previous studies, which neglected the spectrum of the severity of metabolic abnormalities. For the first time, we investigated the number of cumulative MetS components and UC risk to determine a biological gradient for the relationship of MetS with UC. We found that as the number of MetS components increased, the UC risk was elevated and coexistence was more likely. In addition, compared with those with none of the risk factors of MetS, MetS is more common in NMIBC patients.

The mechanisms responsible for the association of MetS with UC are not fully clear. Some studies have indicated the interactions we studied, while the signaling pathways remain unknown. One possible explanation is that the incidence of urinary tract infections is remarkably increased among diabetic subjects, particularly in women, which was considered a risk factor contributing to UC carcinogenesis [27]. Insulin resistance and chronic inflammation are also culprits in the link between obesity and UC [28].

Our study also implicates another clinical factor. It is well known that MetS conveys a pivotal contribution to an increased CAD risk, which remains a leading cause of health impairment [7, 29]. Because MetS plays an important role in CAD, and possibly in UC development, we believe that a deeper understanding of the underlying mechanisms and better control of MetS may obtain more and unexpected benefits.

Our study has several limitations. Although we adjusted for many possible confounders, we did not adjust for other possible residual confounders, such as physical activity and dietary factors. Likewise, information including the duration and treatment of MetS, which may theoretically influence the natural development of UC, was also lacking. In addition, our study was retrospective, which introduces an intrinsic selection bias. This retrospective design only allowed us to examine the temporal relationship between MetS and UC; thereby, causal inferences are limited. Clearly, prospective larger-scale studies are needed.

## Conclusions

MetS was significantly associated with a risk of UC in female patients. Diabetes and hypertriglyceridemia were independent UC risk factors for UC risk. Furthermore, as the number of MetS components increased, the risk of overall UC, NMIBC, and IBC was elevated.

## References

[1] Jemal A, Bray F, Center MM, Ferlay J, Ward E, Forman D (2011). Global cancer statistics. CA Cancer J Clin.

[2] Murta-Nascimento C, Schmitz-Dräger BJ, Zeegers MP, Steineck G, Kogevinas M, Real FX (2007). Epidemiology of urinary bladder cancer: from tumor development to patient’s death. World J Urol.

[3] Freedman ND, Silverman DT, Hollenbeck AR, Schatzkin A, Abnet CC (2011). Association between smoking and risk of bladder cancer among men and women. JAMA.

[4] Li N, Yang L, Zhang Y, Zhao P, Zheng T, Dai M (2011). Human papillomavirus infection and bladder cancer risk: a meta-analysis. J Infect Dis.

[5] Gorbachinsky I, Akpinar H, Assimos DG (2010). Metabolic syndrome and urologic diseases. Rev Urol.

[6] Grundy SM, Cleeman JI, Daniels SR, Donato KA, Eckel RH, Franklin BA (2005). Diagnosis and management of the metabolic syndrome: an American Heart Association/National Heart, Lung, and Blood Institute scientific statement. Circulation.

[7] Alberti KG, Eckel RH, Grundy SM, Zimmet PZ, Cleeman JI, Donato KA (2009). Harmonizing the metabolic syndrome: a joint interim statement of the International Diabetes Federation Task Force on Epidemiology and Prevention; National Heart, Lung, and Blood Institute; American Heart Association; World Heart Federation; International Atherosclerosis Society; and International Association for the Study of Obesity. Circulation.

[8] Russo A, Autelitano M, Bisanti L (2008). Metabolic syndrome and cancer risk. Eur J Cancer.

[9] Cowey S, Hardy RW (2006). The metabolic syndrome: a high-risk state for cancer?. Am J Pathol.

[10] Giovannucci E (2007). Metabolic syndrome, hyperinsulinemia, and colon cancer: a review. Am J Clin Nutr.

[11] Stocks T, Rapp K, Bjorge T, Manjer J, Ulmer H, Selmer R (2009). Blood glucose and risk of incident and fatal cancer in the metabolic syndrome and cancer project (Me-Can): analysis of six prospective cohorts. PLoS Med.

[12] Esposito K, Chiodini P, Colao A, Lenzi A, Giugliano D (2012). Metabolic syndrome and risk of cancer: a systematic review and meta-analysis. Diabetes Care.

[13] Ha¨ggstro¨m C, Stocks T, Rapp K, Bjørge T, Lindkvist B, Concin H (2011). Metabolic syndrome and risk of bladder cancer: prospective cohort study in the metabolic syndrome and cancer project (Me-Can). Int J Cancer.

[14] Ozbek E, Otunctemur A, Dursun M, Koklu I, Sahin S, Besiroglu H (2014). Association between the metabolic syndrome and high tumor grade and stage of primary urothelial cell carcinoma of the bladder. Asian Pac J Cancer Prev.

[15] Cantiello F, Cicione A, Autorino R, Salonia A, Briganti A, Ferro M (2014). Visceral obesity predicts adverse pathological features in urothelial bladder cancer patients undergoing radical cystectomy: a retrospective cohort study. World J Urol.

[16] The Chinese medical association diabetes, metabolic syndrome research consortium (2004). The Chinese medical association advice about metabolic syndrome complications. Chin J Diabetes.

[17] Wallner LP, Morgenstern H, McGree ME, Jacobson DJ, Sauver JLS, Jacobsen SJ (2010). The effects of metabolic conditions on prostate cancer incidence over 15 years of follow-up: results from the Olmsted county study. BJU Int.

[18] Holick CN, Giovannucci EL, Stampfer MJ, Michaud DS (2007). Prospective study of body mass index, height, physical activity and incidence of bladder cancer in US men and women. Int J Cancer.

[19] Koebnick C, Michaud D, Moore SC, Park Y, Hollenbeck A, Ballard-Barbash R (2008). Body mass index, physical activity, and bladder cancer in a large prospective study. Cancer Epidemiol Biomarkers Prev.

[20] Chromecki TF, Cha EK, Fajkovic H, Rink M, Ehdaie B, Svatek RS (2013). Obesity is associated with worse oncological outcomes in patients treated with radical cystectomy. BJU Int.

[21] Goldbohm R, Balder H, Bosch L, Preller L, Schouten L, Brandt P (2006). Is body weight associated with risk of bladder cancer?. Proc Am Assoc Cancer Res.

[22] Nakata S, Sato J, Ohtake N, Lmai K, Yamanaka H (1995). Epidemiological study of risk factors for bladder cancer. Hinyokika Kiyo.

[23] Inoue M, Iwasaki M, Otani T, Sasazuki S, Noda M, Tsugane S (2006). Diabetes mellitus and the risk of cancer: results from a large-scale population-based cohort study in Japan. Arch Intern Med.

[24] Coughlin SS, Calle EE, Teras LR, Petrelli J, Thun MJ (2004). Diabetes mellitus as a predictor of cancer mortality in a large cohort of US adults. Am J Epidemiol.

[25] Larsson SC, Orsini N, Brismar K, Wolk A (2006). Diabetes mellitus and risk of bladder cancer: a meta-analysis. Diabetologia.

[26] Schatzkin A, Hoover RN, Taylor PR, Ziegler RG, Carter CL, Albanes D (1988). Site-specific analysis of total serum cholesterol and incident cancer in the National Health and Nutrition Examination Survey I epidemiologic follow-up study. Cancer Res.

[27] Joshi N, Caputo GM, Weitekamp MR, Karchmer AW (1999). Infections in patients with diabetes mellitus. N Engl J Med.

[28] Kruijsdijk RCM, Wall E, Visseren FL (2009). Obesity and cancer: the role of dysfunctional adipose tissue. Cancer Epidemiol Biomarkers Prev.

[29] Ninomiya JK, L’Italien G, Criqui MH, Whyte JL, Gamst A, Chen RS (2004). Association of the metabolic syndrome with history of myocardial infarction and stroke in the Third National Health and Nutrition Examination Survey. Circulation.

